# Treatment-Associated Differences in Serum Cytokine Profiles in Canine Atopic Dermatitis: A Cross-Sectional Study

**DOI:** 10.3390/ani16152390

**Published:** 2026-08-03

**Authors:** Jae-Yun Ko, Min-Hee Kang, Kwang-Sup Lee, Hee-Myung Park

**Affiliations:** 1 Pyeonanhan Animal Hospital, Daejeon 34069, Republic of Korea; musoi1@naver.com; 2 Department of Veterinary Internal Medicine, College of Veterinary Medicine, Konkuk University, Seoul 05029, Republic of Korea; 3 Department of Bio-Animal Health, Jangan University, Hwaseong 18331, Republic of Korea; mhkang@jangan.ac.kr; 4 Gyeonggi Animal Medical Center, Yongin 17023, Republic of Korea; deu02181@naver.com

**Keywords:** CADESI-04, canine atopic dermatitis, IL-31, pruritus, serum cytokines, systemic immunomodulators

## Abstract

Systemic immunomodulatory treatments are widely used in canine atopic dermatitis (cAD), but treatment-associated differences in circulating cytokine profiles remain unclear. This cross-sectional study compared serum cytokine concentrations in 143 dogs: Healthy (*n* = 28), Untreated AD (*n* = 27), Prednisolone (*n* = 23), Oclacitinib (*n* = 29), Lokivetmab (*n* = 19), and Cyclosporine (*n* = 17). Serum concentrations of IFN-γ, IL-10, IL-13, IL-31, and TGF-β1 were quantified using enzyme-linked immunosorbent assays. pVAS and CADESI-04 were compared among the five cAD groups. All five cytokines differed significantly among the six groups. The Prednisolone group had lower pVAS scores than the Untreated AD group (*p* = 0.002) and the Cyclosporine group (*p* = 0.001), despite having the highest median serum IL-31 concentration (163.9 pg/mL), which was significantly higher than that in each of the other groups (*p* ≤ 0.003). The Prednisolone group showed lower IL-13 concentrations than the Healthy and Untreated AD groups (both *p* = 0.001). IFN-γ concentrations were lower in the Oclacitinib, Lokivetmab, and Cyclosporine groups than in the Healthy group (*p* ≤ 0.002). Serum cytokine profiles differed among groups defined by treatment status; however, these cross-sectional differences cannot be interpreted as direct treatment effects. The discordance between pVAS and serum IL-31 in the Prednisolone group indicates that circulating cytokine concentrations may not consistently parallel concurrent clinical severity and should be interpreted as a hypothesis-generating observation rather than an established biological relationship. Longitudinal studies are needed to clarify whether these profiles reflect treatment exposure, underlying disease heterogeneity, or both.

## Simple Summary

Canine atopic dermatitis is a common long-term skin disease that causes itching and skin inflammation in dogs. Several medicines, including prednisolone, oclacitinib, lokivetmab, and cyclosporine, are used to control these signs. However, it is not fully understood whether dogs receiving these treatments show different immune-related patterns in the blood. We therefore compared five serum cytokines in 143 dogs, including healthy controls, untreated dogs with atopic dermatitis, and dogs receiving different systemic treatments. The treatment groups showed different serum cytokine patterns. Notably, dogs receiving prednisolone had the highest blood concentration of IL-31, a cytokine associated with itching, despite having lower itching scores than some other groups. This finding suggests that blood cytokine concentrations may not always match the severity of itching assessed on the same visit. These results may help veterinarians interpret treatment-associated immune patterns in dogs with atopic dermatitis.

## 1. Introduction

Canine atopic dermatitis (cAD) is a prevalent, relapsing inflammatory dermatosis characterized by pruritus and erythema. Its pathogenesis is complex and multifactorial, involving immune dysregulation, epidermal barrier dysfunction, and hypersensitivity to environmental allergens [1,2].

Type 2 immune responses are considered important contributors to the disruption of inflammatory homeostasis in cAD, particularly in acute and active skin lesions. Among the cytokines implicated in these pathways, interleukin (IL)-31 is closely associated with pruritus, whereas IL-13 may contribute to epidermal barrier dysfunction and cutaneous inflammation [3,4,5,6]. IL-31 is known to promote pruritus through JAK–STAT-dependent signaling in sensory neurons, while IL-13 has been implicated in allergic inflammation and impairment of epidermal barrier function [5,6].

Other cytokines, including IL-10, transforming growth factor-β1 (TGF-β1), and interferon-γ (IFN-γ), have also been investigated in cAD because of their potential roles in immune regulation, inflammatory balance, and disease heterogeneity [7,8,9]. IL-10 and TGF-β1 are key regulatory cytokines and may reflect aspects of regulatory T-cell activity; however, their specific roles in cAD remain unclear because findings across studies have been inconsistent [9]. IFN-γ, a representative Th1-associated cytokine, may also contribute to chronic or heterogeneous immune responses in cAD, although its detection in serum can be challenging because of low basal circulating concentrations [4,10].

Several systemic immunomodulatory therapies are used to control pruritus and skin inflammation in dogs with cAD. Prednisolone exerts broad anti-inflammatory and immunosuppressive effects, whereas cyclosporine suppresses T-cell activation through inhibition of calcineurin-dependent signaling and reduced cytokine production, including IL-2 [11,12]. Oclacitinib preferentially inhibits JAK1-dependent cytokine signaling involved in pruritus, allergic responses, and inflammation, whereas Lokivetmab directly targets canine IL-31 through a species-specific monoclonal antibody mechanism [13,14].

Despite their widespread clinical use, comparative data describing serum cytokine profiles among dogs receiving different systemic immunomodulatory treatments are scarce [15,16,17]. This issue is clinically relevant because serum cytokine concentrations may not fully reflect the complex immunological processes occurring in the skin and other peripheral tissues [18].

Therefore, this cross-sectional study compared serum concentrations of IFN-γ, IL-10, IL-13, IL-31, and TGF-β1 among healthy dogs, untreated AD, and systemic immunomodulatory treatment groups. We hypothesized that treatment groups would show distinct serum cytokine profiles that might not fully correspond with concurrent clinical severity.

## 2. Materials and Methods

### 2.1. Study Population and Diagnostic Criteria

The study population comprised 143 client-owned dogs, including 28 dogs in the Healthy group and 115 dogs diagnosed with cAD. Dogs in the Healthy group had no previous history of dermatological disease and no clinically relevant abnormalities on physical examination.

The diagnosis of cAD required fulfillment of at least five of the eight Favrot’s criteria [19]. Before enrollment, other causes of pruritus, such as superficial pyoderma, ectoparasite infestation, and dermatophytosis, were ruled out. Dogs with cAD received regular ectoparasite prevention and had undergone a dietary elimination trial lasting at least 8 weeks before inclusion.

### 2.2. Classification of Experimental Groups

Dogs were assigned to six groups based on systemic medication use at blood collection. The Healthy group (*n* = 28) comprised dogs without cAD. The Untreated AD group (*n* = 27) included dogs diagnosed with cAD that had received no systemic immunomodulatory treatment during the 4 weeks preceding sample collection.

The Prednisolone group (*n* = 23) comprised dogs receiving oral prednisolone (Solondo; Yuhan, Seoul, Republic of Korea) at 0.25–0.5 mg/kg twice daily for 2–4 weeks. The Oclacitinib group (*n* = 29) included dogs receiving oral oclacitinib (Apoquel; Zoetis, Kalamazoo, MI, USA) at 0.4–0.6 mg/kg once daily for at least 2 weeks. Dogs in the Lokivetmab group (*n* = 19) had received subcutaneous lokivetmab (Cytopoint; Zoetis, Lincoln, NE, USA) according to the manufacturer’s recommended dosing regimen, with the most recent administration occurring within 8 weeks before sampling. The Cyclosporine group (*n* = 17) comprised dogs receiving oral cyclosporine (Sandimmun Neoral; Novartis, Basel, Switzerland) at 3.3–6.7 mg/kg once daily for more than 4 weeks. Treatment selection was determined by the attending clinician according to clinical need. Dogs were excluded if they had received systemic immunomodulatory drugs other than the treatment defining their assigned group, or antihistamines, within 4 weeks before blood collection.

### 2.3. Ethical Considerations

Ethical approval for this study was granted by the Institutional Animal Care and Use Committee of Konkuk University (IACUC Approval No. VNG-25-002-1). Enrollments include privately owned dogs evaluated at veterinary clinic in Daejeon, Republic of Korea, from April through November 2025. Written consent was provided by each dog’s owner before inclusion. To minimize additional discomfort or stress, serum samples were obtained from residual blood collected during routine clinical diagnostic procedures. No blood samples were collected solely for the purposes of this study, and no additional invasive procedures were performed.

### 2.4. Blood Collection, Serum Processing and Storage

During routine clinical assessment, jugular venous blood was obtained and placed in serum-separator tubes, where it was left to clot for 30 min at room temperature. After centrifugation at 3000 rpm for 15 min, the serum fraction was collected. Samples with visible hemolysis or lipemia were excluded. Serum aliquots were placed in cryovials and initially maintained at −20 °C. Within 2 weeks after collection, all aliquots were moved to a −80 °C freezer and stored until cytokine analysis. Each sample was subjected to a single freeze–thaw cycle before analysis.

### 2.5. Evaluation of Pruritus and Skin Lesion Severity

Clinical severity was evaluated using the owner-assessed pruritus visual analog scale (pVAS) for pruritus severity and the clinician-assessed Canine Atopic Dermatitis Extent and Severity Index-04 (CADESI-04) for skin lesion severity [20,21]. The pVAS ranges from 0, indicating no pruritus, to 10, indicating the most severe pruritus. The total CADESI-04 score was obtained by assessing erythema, lichenification, and alopecia/excoriation at 20 predefined body sites and summing the individual scores. The pVAS and CADESI-04 scores were compared among the cAD groups to characterize concurrent pruritus and lesion severity according to treatment status.

### 2.6. Measurement of Cytokines

Serum concentrations of five cytokines were measured using commercially available ELISA kits: IFN-γ (CAIF00; R&D Systems, Minneapolis, MN, USA), IL-10 (MBS450823; MyBioSource, San Diego, CA, USA), IL-13 (CI0043; NeoBiolab, Cambridge, MA, USA), IL-31 (CI0041; NeoBiolab, Cambridge, MA, USA), and TGF-β1 (USEA124CA; USCN, Wuhan, China). Each assay was conducted in accordance with the respective manufacturers’ protocol, following standard ELISA methods previously applied to canine serum samples [22]. Sandwich ELISAs were used for IFN-γ, IL-10, and TGF-β1, whereas competitive ELISAs were used for IL-13 and IL-31. Absorbance was measured at 450 nm, and cytokine concentrations were calculated from the respective standard curves. All samples and standards were measured once, and the obtained values were used for statistical analysis.

### 2.7. Statistical Analysis

Cytokine concentrations were summarized as mean ± SD and median values. Differences in serum cytokine concentrations among the six groups (Healthy, Untreated AD, Prednisolone, Oclacitinib, Lokivetmab, and Cyclosporine) were evaluated with the Kruskal–Wallis test. When significant differences among groups were detected, post hoc pairwise comparisons were conducted with the Mann–Whitney U test with Bonferroni correction. Because 15 pairwise comparisons were possible across the six groups, statistical significance for pairwise comparisons was set at *p* < 0.0033.

pVAS and CADESI-04 were compared among the five cAD groups (Untreated AD, Prednisolone, Oclacitinib, Lokivetmab, and Cyclosporine) using the Kruskal–Wallis test. The Healthy group was excluded from these analyses because pVAS and CADESI-04 were evaluated only in dogs with cAD. When significant differences among groups were detected, post hoc pairwise comparisons were conducted with the Mann–Whitney U test with Bonferroni correction. Because 10 pairwise comparisons were possible across the five cAD groups, statistical significance for pairwise comparisons was set at *p* < 0.005.

For omnibus tests, statistical significance was defined as *p* < 0.05. Statistical analyses were conducted with IBM SPSS Statistics 22.0 for Windows. (IBM Corp., Armonk, NY, USA).

## 3. Results

### 3.1. Characteristics of the Study Groups

The study population comprised 143 dogs distributed across six groups: Healthy (*n* = 28), Untreated AD (*n* = 27), Prednisolone (*n* = 23), Oclacitinib (*n* = 29), Lokivetmab (*n* = 19), and Cyclosporine (*n* = 17) (Table 1).

The Healthy, Untreated AD, Prednisolone, Oclacitinib, Lokivetmab, and Cyclosporine groups had mean ages of 6.93 ± 3.34, 8.44 ± 3.79, 6.21 ± 3.38, 8.55 ± 3.03, 6.57 ± 3.99, and 8.80 ± 2.67 years, respectively.

Table 1 summarizes the distribution of sex and neuter status. The Healthy group comprised 17 males and 11 females, whereas the Untreated AD and Lokivetmab groups consisted exclusively of neutered dogs.

### 3.2. Comparison of Serum Cytokine Levels Across the Six Groups

The levels of all five cytokines differed significantly among the six groups (Table 2). Significant overall group differences were identified for IFN-γ (*p* < 0.001), IL-10 (*p* = 0.001), IL-13 (*p* = 0.001), IL-31 (*p* < 0.001), and TGF-β1 (*p* = 0.001).

**Figure 1:**
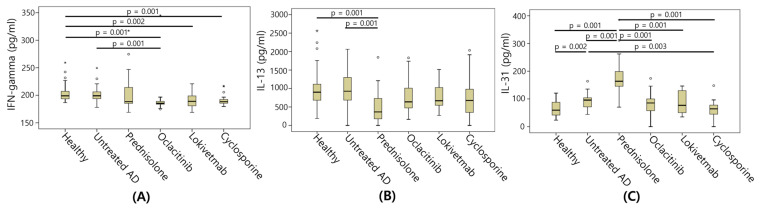
Box-and-whisker plots show differences in serum cytokine concentrations among Healthy, Untreated AD, Prednisolone, Oclacitinib, Lokivetmab, and Cyclosporine groups. Box-and-whisker plots show the serum concentrations of IFN-γ (**A**), IL-13 (**B**), and IL-31 (**C**) across the six groups. The central horizontal line inside each box marks the median, while the lower and upper box boundaries indicate the 25th and 75th percentiles, respectively. Circles and asterisks indicate outliers. AD: Atopic dermatitis; IFN-γ: Interferon-gamma; IL-13: Interleukin-13; IL-31: Interleukin-31.

The Healthy group showed significantly higher IFN-γ concentrations than the Oclacitinib (*p* = 0.001), Lokivetmab (*p* = 0.002), and Cyclosporine groups (*p* = 0.001). The Untreated AD group also showed higher IFN-γ concentrations than the Oclacitinib group (*p* = 0.001) (Figure 1A). The Cyclosporine group showed significantly lower IL-10 concentrations than the Prednisolone (*p* = 0.002), Oclacitinib (*p* = 0.001), and Lokivetmab groups (*p* = 0.002).

The Prednisolone group had significantly lower IL-13 concentrations than the Healthy and Untreated AD groups (both *p* = 0.001) (Figure 1B). Among the six groups, the highest IL-31 level was found in the Prednisolone group. This concentration was significantly greater compared with the concentrations in the Healthy (*p* = 0.002), Untreated AD (*p* = 0.001), Oclacitinib (*p* = 0.001), Lokivetmab (*p* = 0.001), and Cyclosporine groups (*p* = 0.003). In addition, the Untreated AD group had significantly higher IL-31 concentrations than the Healthy and Cyclosporine groups (both *p* = 0.001) (Figure 1C). The Lokivetmab group had significantly lower TGF-β1 concentrations than the Prednisolone and Oclacitinib groups (both *p* = 0.001).

### 3.3. Comparison of pVAS and CADESI-04 Scores Among the cAD Groups

Both pVAS and CADESI-04 differed significantly among the five cAD groups (both *p* < 0.001).

**Figure 2:**
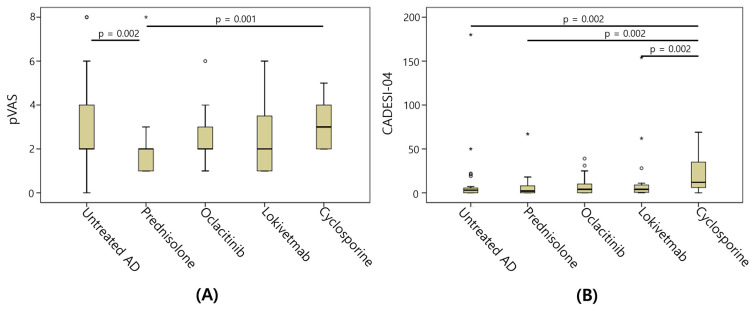
Box-and-whisker plots comparing pVAS (**A**) and CADESI-04 (**B**) scores among the five cAD groups. The central horizontal line inside each box marks the median, while the lower and upper box boundaries indicate the 25th and 75th percentiles, respectively. The whiskers indicate the range excluding outliers. Circles and asterisks indicate outliers. Horizontal bars indicate significant pairwise comparisons based on the Mann–Whitney U test with a Bonferroni-adjusted significance threshold of *p* < 0.005. pVAS: Pruritus visual analog scale; CADESI-04: Canine Atopic Dermatitis Extent and Severity Index-04; AD: Atopic dermatitis.

For pVAS, the Prednisolone group had significantly lower scores than the Untreated AD group (*p* = 0.002) and the Cyclosporine group (*p* = 0.001). No other pairwise comparisons reached the Bonferroni-adjusted significance threshold (Figure 2A).

For CADESI-04, the Cyclosporine group had significantly higher scores than the Untreated AD, Prednisolone, and Oclacitinib groups (all *p* = 0.002). No other pairwise comparisons reached the Bonferroni-adjusted significance threshold (Figure 2B).

## 4. Discussion

This cross-sectional study compared serum concentrations of IFN-γ, IL-10, IL-13, IL-31, and TGF-β1 among healthy dogs, untreated AD, and dogs receiving different systemic immunomodulatory treatments. Serum cytokine patterns differed among groups defined by treatment status. The most notable finding was that the Prednisolone group had the highest serum IL-31 concentrations despite lower pVAS scores than the Untreated AD and Cyclosporine groups. These results should be interpreted cautiously as associations rather than direct effects of treatment, because treatment allocation was based on clinical need and the cross-sectional design does not permit causal inference.

IL-31 is a key cytokine involved in pruritus in cAD [10]. In the present study, the Prednisolone group showed the highest serum IL-31 concentration despite having lower pVAS scores than the Untreated AD and Cyclosporine groups. A previous study reported only a weak correlation between serum IL-31 concentrations and pVAS in dogs with cAD [15]. In addition, serum IL-31 was undetectable in a substantial proportion of dogs with chronic cAD despite the presence of pruritus [5]. This group-level discordance indicates that serum IL-31 concentrations may not consistently parallel contemporaneous pruritus severity across treatment groups. This finding should be interpreted as a hypothesis-generating observation rather than as evidence of persistent cutaneous inflammation or sustained IL-31 signaling because neither skin cytokine expression nor receptor-mediated signaling was assessed. Differences in treatment duration, corticosteroid dose, disease phenotype, timing of blood collection, and individual pharmacodynamic responses may have contributed to the observed pattern. However, these factors were not directly evaluated in this study. The lower IL-31 concentration in the Cyclosporine group relative to the Untreated AD group may be compatible with the broad T-cell suppressive effects of cyclosporine; however, this interpretation remains tentative because of the cross-sectional design and potential differences in baseline disease characteristics. In human patients with atopic dermatitis, serum IL-31 concentrations decreased after cyclosporine treatment; however, whether a similar response occurs in dogs remains unclear [23]. The IL-31 findings in the Lokivetmab group also require cautious interpretation. The interval between the most recent lokivetmab administration and blood sampling varied within the permitted 8-week window. Therefore, the observed IL-31 concentration in this group may reflect not only biological variation but also differences in sampling timing [24].

Serum IL-13 concentrations did not differ significantly between the Healthy and Untreated AD groups, whereas the Prednisolone group had lower IL-13 concentrations than both groups. IL-13 has been implicated in type 2 inflammation, epidermal barrier dysfunction, and allergic skin disease [4,6]. The lower IL-13 concentration in the Prednisolone group could be related to the broad anti-inflammatory and immunosuppressive actions of glucocorticoids. Prednisolone has been reported to suppress cutaneous IL-13 expression in an experimental canine late-phase reaction model [11]. In addition, decreased serum IL-13 concentrations have been reported after treatment with prednisolone or oclacitinib in dogs with spontaneous cAD [25]. However, differences in biological samples, disease models, and treatment protocols limit direct comparison with the present findings. No significant difference in IL-13 concentration was identified between the Cyclosporine, Oclacitinib, or Lokivetmab groups and the Untreated AD group after correction for multiple comparisons. Accordingly, the present results do not support definitive conclusions regarding the effects of these treatments on circulating IL-13 concentrations.

IFN-γ is a representative Th1-associated cytokine, and previous studies of serum IFN-γ in cAD have reported inconsistent findings, including no difference between healthy and affected dogs, concentrations below the detection limit, and lower concentrations in dogs with cAD [4,26,27]. In the present study, IFN-γ concentrations did not differ significantly between the Healthy and Untreated AD groups. However, IFN-γ concentrations were lower in the Oclacitinib, Lokivetmab, and Cyclosporine groups than in the Healthy group, and the Oclacitinib group also showed lower concentrations than the Untreated AD group. These findings may reflect differences in systemic cytokine profiles associated with treatment status, differences in disease characteristics among treatment groups, or both. The immunomodulatory actions of prednisolone, oclacitinib, and cyclosporine may be relevant when interpreting the observed cytokine patterns [28,29]. In contrast, evidence that lokivetmab directly alters IFN-γ production remains limited [30]. The lower IFN-γ concentration in the Lokivetmab group should therefore not be attributed to a direct drug effect without longitudinal evidence.

Serum IL-10 concentrations were lower in the Cyclosporine group than in the Prednisolone, Oclacitinib, and Lokivetmab groups. Cyclosporine inhibits calcineurin-dependent T-cell activation and can influence the production of several cytokines, including regulatory cytokines [31]. Nevertheless, the lower IL-10 concentration observed in the Cyclosporine group cannot be separated from possible confounding by indication. This group also had higher CADESI-04 scores than the Untreated AD, Prednisolone, and Oclacitinib groups, suggesting that disease chronicity, lesion severity, treatment selection, or other unmeasured clinical characteristics may have contributed to the observed cytokine profile. Previous studies have reported variable IL-10 findings in dogs with cAD, and no consistent interpretation of circulating IL-10 has been established [17,32].

TGF-β1 concentrations were lower in the Lokivetmab group than in the Prednisolone and Oclacitinib groups. TGF-β1 is produced by multiple immune and nonimmune cell types and may be influenced by disease activity, tissue injury, regulatory immune responses, treatment exposure, and analytical conditions [33,34,35]. Therefore, this difference should not be interpreted as evidence that prednisolone or oclacitinib increased, maintained, or failed to suppress systemic TGF-β1 activity. Data regarding the association between oclacitinib treatment and circulating TGF-β1 remain limited, and previous reports have not consistently demonstrated changes in serum TGF-β1 after treatment [17]. Further longitudinal studies using standardized sampling intervals are required to clarify the clinical relevance of these findings.

Several limitations should be considered. Because all samples and standards were measured once, intra-assay coefficients of variation could not be calculated. This may have reduced the reliability of the absolute cytokine concentrations, which should therefore be interpreted cautiously. Potential ELISA batch-to-batch variation was not specifically evaluated and therefore cannot be excluded. The wide variability observed in some cytokine concentrations may reflect biological heterogeneity, differences in disease characteristics, or analytical variability; however, the source of this variability could not be determined in the present study. In addition, age, sex and neuter status, disease duration and clinical severity were not included as covariates. Therefore, potential confounding by these factors cannot be excluded. The relatively small and unequal group sizes may also have limited statistical power and the ability to adjust for potential confounding factors. The cross-sectional design precluded within-dog assessment of cytokine changes before and after treatment and did not permit causal inference. In addition, treatment selection was determined by clinical need rather than random allocation, introducing the possibility of confounding by indication. Differences in disease duration, lesion chronicity, prior treatment history, severity at treatment initiation, and other clinical characteristics may therefore have influenced both treatment selection and serum cytokine concentrations. Furthermore, treatment duration, drug dose, and the interval between treatment administration and blood collection were not fully standardized, which may have been particularly relevant for oclacitinib and lokivetmab given their differing pharmacokinetic and pharmacodynamic profiles [24,36]. Therefore, the observed differences among the groups cannot be interpreted as direct treatment effects. It should also be noted that serum cytokine concentrations may not directly reflect cytokine expression or signaling within lesional skin.

Overall, the observed differences in serum cytokine concentrations among groups defined by treatment status warrant further investigation. Longitudinal studies incorporating standardized treatment protocols, serial serum sampling, and paired skin-based analyses are needed to determine whether these circulating cytokine patterns reflect treatment exposure, underlying disease heterogeneity, or both.

## 5. Conclusions

Serum cytokine profiles differed among the cAD groups defined by treatment status. Notably, the Prednisolone group had the highest serum IL-31 concentrations despite lower pVAS scores than the Untreated AD and Cyclosporine groups, indicating that circulating IL-31 may not consistently parallel concurrent pruritus severity across treatment groups. This finding should be interpreted as hypothesis-generating rather than as an established biological relationship as variation in blood collection timing, drug dose, and disease severity may have contributed to this observation. Differences in IFN-γ, IL-10, IL-13, and TGF-β1 were also observed; however, these findings should be interpreted as preliminary observations rather than direct treatment effects. Longitudinal studies with standardized treatment protocols and serial sampling are needed to clarify the clinical relevance of these cytokine patterns.

## Figures and Tables

**Table 1:** Characteristics of dogs in this study.

Variable		Healthy	AD				
			Untreated AD	Prednisolone	Oclacitinib	Lokivetmab	Cyclosporine
Number	*N*	28	27	23	29	19	17
	%	20	19	16	20	13	12
Age	Mean	6.93	8.44	6.21	8.55	6.57	8.80
	SD	3.34	3.79	3.38	3.03	3.99	2.67
Sex	Intact male	7	0	1	2	0	0
	Intact female	3	0	2	4	0	5
	Castrated male	10	20	10	17	14	6
	Spayed female	8	7	10	6	5	6

AD: Atopic dermatitis; N: Number; SD: Standard deviation.

**Table 2:** Comparison of serum cytokine levels across the six study groups.

Variable	Healthy	Untreated AD	Prednisolone	Oclacitinib	Lokivetmab	Cyclosporine	*p*
	Mean ± SD	Mean ± SD	Mean ± SD	Mean ± SD	Mean ± SD	Mean ± SD	
IFN-γ	204.78 ± 17.23	201.76 ± 15.34	204.23 ± 36.07	190.87 ± 30.13	190.07 ± 15.31	191.67 ± 11.23	<0.001
IL-10	99.32 ± 173.20	49.08 ± 4.93	133.06 ± 336.74	64.62 ± 35.29	86.44 ± 146.10	46.61 ± 2.38	0.001
IL-13	1036.46 ± 557.43	1002.21 ± 444.57	505.20 ± 475.01	785.63 ± 447.87	789.70 ± 350.21	774.66 ± 575.16	0.001
IL-31	64.37 ± 28.97	91.02 ± 27.15	179.29 ± 70.62	78.24 ± 42.78	87.28 ± 40.33	59.25 ± 37.55	<0.001
TGF-β1	34.34 ± 5.41	35.78 ± 7.47	37.62 ± 6.95	37.08 ± 6.23	32.96 ± 3.41	33.40 ± 3.99	0.001

AD: Atopic dermatitis; SD: Standard deviation; IFN-γ: Interferon-gamma; IL-10: Interleukin-10; IL-13: Interleukin-13; IL-31: Interleukin-31; TGF-β1: Transforming growth factor-beta1. *p*-values were obtained using the Kruskal–Wallis test.

## Data Availability

The data presented in this study are available on request from the corresponding author due to privacy and ethical restrictions related to case-level clinical data collected from client-owned animals.
